# Mediating Effect of Cognitive Social Capital on the Relationship Between Physical Disability and Depression in Elderly People of Rural Pakistan

**DOI:** 10.3390/ijerph16214232

**Published:** 2019-10-31

**Authors:** Azam Tariq, Tian Beihai, Sajjad Ali, Nadeem Abbas, Aasir Ilyas

**Affiliations:** 1 Department of Sociology, College of Humanities and Social Sciences, Huazhong Agricultural University, Wuhan 430070, China; azam_tariq@webmail.hzau.edu.cn; 2 College of Economics and Management, Huazhong Agricultural University, Wuhan 430070, China; sajjad@webmail.hzau.edu.cn (S.A.); aasirilyas@yahoo.com (A.I.); 3 Institute of Social & Cultural Studies, University of the Punjab, Lahore 54000, Pakistan; nadeem544abbas@gmail.com

**Keywords:** cognitive social capital, interpersonal trust, reciprocity, physical disability, depression, elderly people

## Abstract

Cognitive social capital is crucial for mental wellbeing and physical disability in order to avoid late-life depression. The objective of this study was to investigate the mediating effect of cognitive social capital (interpersonal trust and reciprocity) on the relationship between physical disability and depression in elderly people of rural Pakistan. For this purpose, 146 respondents aged 60 years or above and residents of rural areas of district Muzaffargarh (Punjab, Pakistan) were approached for data collection. The questionnaire includes socio-demographic variables (gender, age, education, marital status, family system, living status, household income, and number of chronic diseases); the geriatric depressive symptoms scale (GDS-15) was used to measure depression, physical disability was evaluated through ADL and IADL scales, and cognitive social capital, which includes interpersonal trust and reciprocity, was measured using single-item questions. It was found that interpersonal trust, reciprocity, depression, and physical disability were significantly correlated with each other and physical disability was directly associated with depression. In mediation analysis, reciprocity mediated the relationship between physical disability and depression. Our findings highlight the need to enhance cognitive social capital interventions and develop policies to promote mental and physical health of rural elderly.

## 1. Introduction

Depression is known as the global mental health problem in elderly people [1]. Meta-analytic research found that 3.29% of elderly population of the world are temporary and 16.52% are permanent victims of depression [2]. Variations in depressive symptoms among elderly people in different societies highlight the need of further research to investigate the effect of social atmosphere with respect to late-life depression [3]. The projections based on prior researches show that depression will be the second foremost burden of disease at global level by the year 2020 [4]. In fact, the majority of elderly people with low socioeconomic status and poor living standard are victims of depression [5,6]. In 2017, the population aged 60 or above in Pakistan was 11.3 million and it is expected to increase up to 43.3 million (almost 15.8% of total population) by 2050 [7]. The prevalence of depression in rural and urban settings ranged 18–66% in Pakistan [8,9]. Recently, attention has been shifted and there is more emphasis on the effect of social environment on the psychological disorders in elderly people [10]. This study attempts to investigate the mediating effect of cognitive social capital on the relationship between physical disability and depression in elderly people living in rural areas of Pakistan. Such exertion will be helpful to both fill the literature gap, specifically for rural elderly of Pakistan, and to develop policies for the elderly population.

Physical disability is known as the inability to perform basic tasks of self-care in daily life, which affect an individual’s association with both physical and social surroundings [11]. The connection between body and mind is a versatile concept and sufficient literature is available highlighting the adjacent relationship between physical impairment and mental disorder [12]. Physical impairment often leads elderly people vulnerable to depression [13,14,15,16]. It has been proved that impairment in activities of daily living (ADL) and instrumental activities of daily living (IADL) are associated with depression [11]. Inability to carry out activities of daily living is a source of depression in elderly people [11,17]. The presence of physical disability causes increased geriatric depressive symptoms [11]. Furthermore, elderly people who are unable to perform ADL have higher levels of depression [17]. 

Social capital is known as an upstream element for mental health and explains the probable variations in mental wellbeing among different societies or nations [4,5,6,7,8,9,10,11,12,13,14,15,16,17,18]. Social capital is known as the structure of social systems or connections, which includes mutual social trust, the standard of reciprocity, and network participation enabling people to contribute in society-based shared benefits [19]. Social capital is further divided into two parts, cognitive and structural social capital. Cognitive social capital refers to the determined social cohesion based on the subjective part of perceived interpersonal trust, reciprocity, and social support. On the other hand, objective quantifiable factors of social participation and social networks are defined as structural social capital [20,21]. Previous research findings show that lower level of social capital in a person or specific community was directly linked with greater mental disorders, including depression and high suicide rate [21,22,23]. Plenty of literature has suggested that social capital is a determinable social factor of public health, especially in elderly people and for normal or prosperous aging [24]. Furthermore, interpersonal trust and reciprocity are the strongest predictors of mental health factors like subjective wellbeing and life satisfaction in elderly people [25]. Building on this association, it has been found that social capital has significant effects on satisfaction of life, self-rated health condition, depression, physical disability, and death rate in the elderly population [26,27,28]. Regardless of methodological problems in social capital investigations, sociologists, policy makers, and international organizations like World Health Organization (WHO) and World Bank are highly interested in accepting and following the perspective that social capital is a major social determinant of cognitive health and contributes to mental wellbeing [29]. The remarkable rise in life expectancy and old age population distribution of the world highlights the need to find out the effect of social capital on depression in elderly people. Almost two-thirds of the elderly population belong to underdeveloped countries [7]. Pakistan is a developing country facing many socio-economic problems and, hence, is not able to develop significant policies to deal with the problems of the elderly population. However, some non-government sector organizations are providing care services to these neglected elderly people. Moreover, there are few studies that have been conducted to explore the effects of social capital on physical disability and depression in elderly population.

A theoretical explanation of the relationship among individuals or distinct levels of cognitive social capital with depressive symptoms might be based on two hypothetical models [30,31]. First, the *main effect model* theorizes that mental health can be protected through living in an extremely trustable community [32]. Thus, when individuals interact or cooperate with neighbors, they may feel a sense of self-worth, belongingness, security, and acceptance within the society or community, and this fact can lead to a positive psycho-social state of an individual [30,31,32]. Individual social capital is an important source of mental health for elderly people [33,34]. Second, the *stress buffering model* theorizes that the adverse emotional and mental reactions to traumatic or stressful situation in life can be prevented or modulated by perceived availability of social capital through careful assessment of circumstances [30]. In addition, physical disability is known as a type of chronic stressor and its presence makes elderly people vulnerable to depression [13,35]. These theoretical models served as the theoretical framework for advancing our research discoveries and might have better implications for maintaining mental health by protecting elderly people from the stressful events of life.

**Figure 1:**
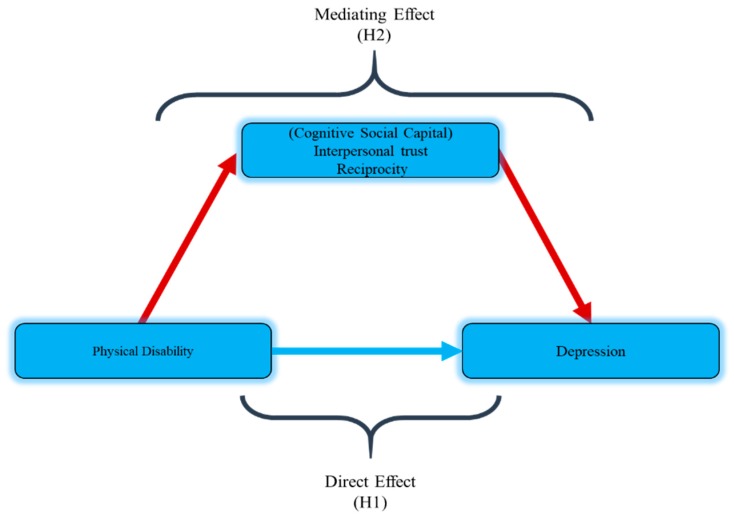
Hypothetical model.

Thus, the present study hypothesized that physical disability (ADL and IADL) is associated with higher depression (H1). This study also hypothesized that cognitive social capital (interpersonal trust, reciprocity) could mediate the relationship between physical disability and depression (H2) among the rural elderly of Pakistan. Figure 1 shows the hypothetical model. 

## 2. Materials and Methods

### 2.1. Study Participants

The respondents for the current study were selected from rural areas of two tehsils (sub-administrative zones under district authority) Alipur and Jatoi of the Muzaffargarh district in the Punjab province of Pakistan through purposive sampling by identifying the people aged 60 years or above (this age is considered to be elderly by the United Nations). A total of 146 participants were approached with the help of Union council Chairman’s (a key person in the rural community). A questionnaire-based interview, translating the questions into the native language, was performed to collect the data from the respondents. The elderly people with extreme physical health problems like terminal illness and severe hearing impairment were not approached for this study. All the participants were informed about the basic goal of this study and they were free to participate or to leave the investigation.

### 2.2. Measurement Variables

#### 2.2.1. Depression

The basic outcome variable depression was measured by using the 15-item geriatric depression scale-short form (GDS-15). GDS is more suitable to measure late-life depression and can be used for both self-administered and interview-based studies [36]. Furthermore, this scale has been used in previous studies on elderly people in Pakistan [37]. It consists of 15 questions with “Yes” and “No” options and depression scores ranged from 0–15. All the responses that showed depression were given a score of 1; otherwise, the score was 0. The higher obtained scores indicate higher depression. This scale was found to be reliable as the Cronbach alpha (α) for this sample was 0.74.

#### 2.2.2. Physical Disability

Physical disability was measured via ADL scale [38] and IADL scale [39]. The ADL scale measures 6 types of basic activities including bathing, dressing, toileting, transferring, and continence, while the IADL scale measures 8 types of more difficult activities, which include using a telephone, shopping, mode of transportation, handling finances, laundry, taking medicine, housekeeping, and food preparation. The data were collected on a four-point Likert scale, and scores ranged from 1 to 4. The maximum score was 56, and higher scores indicate higher physical disability. [11]. The internal consistency Cronbach alpha (α) for the present sample was 0.96.

#### 2.2.3. Cognitive Social Capital

A single-item questionnaire was adopted to measure the cognitive dimensions of social capital, which includes interpersonal trust and reciprocity. 

Question 1. Do you think that most of the people around you are trustworthy?” was used for interpersonal trust, using a 3-point scale: (1) ‘maximum people are trustworthy’, (2) ‘try to be careful’, and (3) ‘I don’t know’.

Question 2. Are you ready to help your neighbor who is in need of your urgent help?” was used to measure reciprocity and was obtained on a 5-point Likert scale ranging from (1) ‘strongly yes’ to (5) ‘strongly no’. Lower obtained scores from a total of five scores presented higher levels of reciprocity. [10].

### 2.3. Analytical Techniques

Statistical package for social sciences (SPSS 21) was used for data analysis. First, descriptive statistics were calculated to find out the distribution of socio-demographic characteristics, which also includes current living status, number of chronic diseases, and prevalence of depression. The mean and standard deviation was calculated for cognitive social capital, physical disability (ADL and IADL), and depression. An independent *t*-test was performed to analyze continuous variables for post hoc multiple comparisons. Pearson correlation coefficient analysis was conducted to find out the correlation among physical disability, cognitive social capital, and depression.

The input or influence of physical disability and cognitive social capital on depression was determined by conducting multiple linear analysis. The mediating effect analysis was conducted via PROCESS model-4 [40], which can be operated through SPSS and other available statistical software packages. In this analysis, the mediation effect of two dimensions of cognitive social capital (interpersonal trust and reciprocity) on depression as an outcome variable was discovered through bootstrapping technique.

## 3. Results

Table 1 shows the socio-demographic characteristics and depression of the respondents. The majority of the respondents (72.6%) were male, whereas female respondents were 37.4%. Moreover, 65.6% of the respondents were below the age of 80 years. As far as the level of education of the respondents is concerned, almost half of the respondents were illiterate, whereas almost 17% to 18% of the respondents had primary, middle, and secondary level of education. Furthermore, 51.3% of the respondents were married. However, 63.0% of the elderly belonged to joint family system and 37% of them were living in nuclear families. Moreover, 53.4% of the research respondents were living with others (son, relatives etc.) while 46.5% were living with their spouse. Furthermore, 43.1% of research participants fell on a low level of income (≤15,000 pkr/month) and the majority (82.8%) of them were suffering from one or two chronic diseases.

Gender and age of the respondents did not have any significant influence on depression. Post hoc multiple comparisons demonstrated that depression was higher in those respondents who were illiterate and had primary-level education. The respondents with single, divorced, or widowed status scored high on geriatric depressive symptoms scale. The participants that belonged to a nuclear family and, interestingly, respondents living with a spouse scored high on geriatric depressive symptoms scale as compared to those who belonged to a joint family system and living with others. Furthermore, the prevalence of depression was higher in respondents with average monthly income of 30,000pkr or less. Lastly, it was found that elderly participants with two or three chronic diseases showed higher depression (Table 1).

Table 2 presents the Pearson correlation coefficient analysis between two dimensions of the cognitive social capital (interpersonal trust, reciprocity), physical disability, and depression. Physical disability was directly correlated with depression (r = 0.249, *p* ˂ 0.01). An inverse correlation was found among interpersonal trust, reciprocity, and depression (r = −0.196, *p* ˂ 0.05 and r = −0.251, *p* ˂ 0.01, respectively). Moreover, from two dimensions of cognitive social capital, reciprocity showed an inverse correlation with physical disability (r = −0.218, *p* ˂ 0.01) indicating that a low level of reciprocity was associated with greater physical disability while interpersonal trust was not found to be significantly correlated with physical disability (r = −0.012, *p* > 0.05).

Multiple linear regression analysis included those socio-demographic variables with a significant influence on depression. Hence, level of education, marital status, family system, living status, average monthly income, and number of chronic diseases were included in the regression analysis. All these confounding variables were controlled in this analysis. Preliminary analyses were performed to ensure there was no violation of the assumption of linearity, normality, and multicollinearity. A significant regression model was found (F = 5.738, *p* ˂ 0.01) with R^2^ of 0.380. The results demonstrated that physical disability was directly associated with depression (β = 0.225, *p* ˂ 0.01) as predicted in hypothesis (H1). Therefore, an increase of one value in standard deviation for physical disability was associated with 0.225 times increase in standard deviation for depression. Likewise, interpersonal trust (β = −0.165, *p* ˂ 0.05) and reciprocity (β = −0.264, *p* ˂ 0.01) were inversely associated with depression. Hence, an increase of one value in standard deviation for interpersonal trust and reciprocity was associated with −0.165 and −0.264 times decrease in standard deviation for depression, respectively; whereas reciprocity (β = −0.264) showed more negative association as compared to interpersonal trust (β = −0.165). Furthermore, unstandardized coefficients showed that one-unit change in physical disability, interpersonal trust, and reciprocity was associated with the amount of 0.08, −0.924, and −1.087 change for depression, respectively (Table 3). 

**Figure 2:**
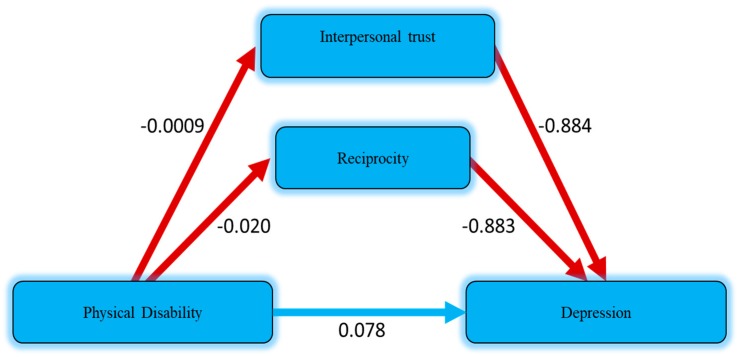
Estimated mediation effect of cognitive social capital on physical disability and depression.

Table 4 presents the mediation effect of cognitive social capital between physical disability and depression. The direct and indirect effect of cognitive social capital and physical disability was analyzed based on model 4 in PROCESS macro (v3.3) created by Preacher and Hayes, which can be accessed from (*http://www.processmacro.org/download.html*). Average monthly income and living status were set as control variables. The current analysis was conducted by means of bootstrapping technique with 5000 resamples. The results (R^2^ = 0.155, 0.224, F = 6.510, 6.715, *p* ˂ 0.05) revealed the significance of the whole model. It was found that physical disability was significantly associated with depression (B = 0.097, *p* ˂ 0.01) without entering cognitive social capital into the equation, which was then mediated (B = 0.078, *p* ˂ 0.01) by interpersonal trust (B = −0.884, *p* ˂ 0.05) and reciprocity (B = −0.883, *p* ˂ 0.01); whereas indirect effect (partial mediation) of reciprocity was 0.017 (−0.020*−0.883). The total effect was 0.095 (0.078 + 0.017); on the other hand, interpersonal trust was not considered to mediate the effect because of its low coefficient value (0.0009). However, overall results were considered to be consistent, as predicted in the second hypothesis H2 that two dimensions of cognitive social capital could mediate the relationship between physical disability and depression in rural elderly people (Figure 2).

## 4. Discussion

In the current study, we investigated the effect of cognitive social capital, which includes interpersonal trust and reciprocity, on the relationship between physical disability and geriatric depression in the elderly population of rural Pakistan. The respondents in this study that had lower levels of education (65% had primary level education or less); were single, divorced, and widowed (49.2% had single, divorced, or widowed status); belong to nuclear family (36.9%); lived with spouse (46.5%); lower level of average income (82.8%) (had average monthly income of ˂30,000 PKR); and with more than one chronic diseases (57.5% had two or three chronic diseases) scored higher levels of depression. These elements all show the individuals socioeconomic status and quality of living. It is suggested by buffering hypothesis that social capital is advantageous for the people with lower socioeconomic status with regard to health [20]. This hypothesis demonstrates that access to social capital is helpful to improve the health of people with lower socioeconomic status by minimizing perceived effects of lower socioeconomic status on health [41].

This study revealed that physical disability was directly associated with depression by confirming previous studies showing the same results as the relationship between limitations in activities of daily living and mental health problems [11]. A study found that lower functioning in activities of daily living predicted higher level of depression among the elderly population [17]. Therefore, poor ADL functioning was the cause of depression found among elderly people [42]. It was determined that physical limitation has a negative effect on mental health [13,15,16,43] and causes psychosocial problems in late years of life [44].

Interpersonal trust and reciprocity were inversely linked with depression. Higher interpersonal trust and reciprocity predicted lower levels of depression. Cross-sectional research was conducted on Chinese elderly (aged 60 years or older) using the geriatric depression scale (long form with 30 items) and it was found that both interpersonal trust and reciprocity were inversely correlated with depression [45]. Similarly, a review of previous studies demonstrated that individual cognitive social capital acts as a resistance against common mental disorders [46]. In 2012, a cross-sectional study with a sample size of 6838 respondents aged 60–80 years found that interpersonal trust and reciprocity were closely associated with symptoms of depression [47]. Furthermore, Korean-based research with 5969 elderly people revealed an indirect relationship between interpersonal trust and reciprocity [10].

Mediating effect analysis included control variables, such as average monthly income and living status of the respondents, which could probably confound the relationship among cognitive social capital, physical disability, and depression. The results show that reciprocity mediated the relationship between physical disability and depression. That is, when the relationship between physical disability and depression was mediated by reciprocity, less physical disability was associated with less depression. Thus, this association is imperative to realize the impact of reciprocity in mitigating the negative effects of physical disability on depression, helps to determine the relative efficacy of cognitive social capital as a mediator on the relationship between physical disability and depression, and has important implications for policy interventions, caregivers, and researchers. Previous studies focused on the direct effect of cognitive social capital and mental health [48] and one study investigated the mediating effect of cognitive social capital on the relationship between socio-economic condition and depressive symptoms in older adults [10]. To the best of our knowledge, this is the first research of its kind, in which we discovered the mediating effect of cognitive social capital on the relationship between physical disability and depression in rural elderly. It demonstrates that individuals with physical disability have greater chances of developing depression in the absence of cognitive social capital and highlights the importance of helping neighborhoods in preventing late-life mental and physical health problems. 

## 5. Conclusions

The present study was conducted to investigate the mediating effect of cognitive social capital on the relationship between physical disability and depression in elderly people living in rural Pakistan. The data were collected through purposive sampling technique with an in-depth interview schedule. The findings of the current study facilitate evidence that cognitive social capital has a passive effect on physical disability and depression among older adults. We found that reciprocity mediates the relationship between physical disability and depression. Furthermore, this study revealed that low levels of interpersonal trust and reciprocity leads to a greater level of depression in elderly people. This suggests that mistrust, lack of help in society, and lower standard of life has inverse effects on physical disability and mental disorders among older people. The findings could be helpful in developing policies and programs for public awareness, to promote and maintain social capital activities to safeguard our elderly people from physical disabilities and late-life mental disorders.

In terms of limitations, the focus of this study on rural community residents may affect the overall situation. Further cross-sectional studies based on large samples and longitudinal studies can be conducted to assess or confirm the associations. However, experimental studies may also be conducted in order to find causality.

## Figures and Tables

**Table 1:** Comparison of different socio-demographic characteristics with depressive symptoms.

Socio-Demographic Variables	No.	Percentage (%)	Depression $\bar{X}\pm \mathrm{SD}$	*F*	*p*
Gender Male Female	106 40	72.6 27.3	8.10 ± 3.43 7.77 ± 3.48	0.264	0.608
Age (Years) 60–69 70–79 80–89 ≥90	49 47 28 22	33.5 32.1 19.1 15.0	7.46 ± 3.06 8.51 ± 3.85 7.64 ± 3.45 8.63 ± 3.23	1.086	0.357
Level of education Illiterate Primary (5 Years) Middle (8 Years) ≥High school	69 26 26 25	47.2 17.8 17.8 17.1	7.23 ± 3.50 9.57 ± 2.91 8.30 ± 3.10 8.24 ± 3.63	3.218	0.025
Marital status Single Married Divorced/Widowed	34 75 38	23.2 51.3 26.0	8.76 ± 3.46 7.20 ± 3.29 8.92 ± 3.40	4.384	0.014
Family system Joint family Nuclear family	92 54	63.0 36.9	7.58 ± 3.29 8.74 ± 3.58	3.904	0.050
Living status Living with a spouse Living with Others (Son or relatives)	68 78	46.5 53.4	8.80 ± 3.51 7.32 ± 3.24	7.085	0.009
Average monthly income (PKR) ≤15,000 15,001–30,000 >30,000	63 58 25	43.1 39.7 17.1	7.87 ± 3.52 8.72 ± 3.29 6.72 ± 3.27	3.184	0.046
No. of chronic diseases 1 2 3	62 59 25	42.4 40.4 17.1	7.00 ± 3.46 8.64 ± 3.27 9.04 ± 3.22	5.064	0.008

**Table 2:** Descriptive statistics and Pearson correlation analysis.

Variables	Range	Mean	SD	Interpersonal Trust	Reciprocity	Depressive Symptoms
Physical disability	14~56	23.58	8.91	−0.012	−0.218 **	0.249 **
Interpersonal trust	1~3	1.50	0.61	-	0.011	−0.196 *
Reciprocity	1~3	1.69	0.83	-	-	−0.251 **
Depressive symptoms	1~14	8.01	3.44	-	-	-

Standard deviation = SD, correlation is significant at 0.05 level = *, correlation is significant at 0.01 level = **.

**Table 3:** Multiple linear regressions on depressive symptoms.

Dependent Variable: Depression	Unstandardized Coefficient	Standardized Coefficient
	B (SE)	β
level of Education (Ref. = Illiterate) Primary (5 Years) Middle (8 Years) ≥High school	1.819 (0.695) 0.988 (0.675) 1.615 (0.703)	0.203 ** 0.110 0.177 *
Marital status (Ref. = Single) Married Divorced/Widowed	−0.606 (0.623) 0.507 (0.705)	−0.088 0.065
Family system	0.951 (0.510)	0.134
Living status	−1.082 (0.504)	−0.157 *
Average monthly income (PKR) (Ref. ≤15,000) 15,001–30,000 >30,000	0.871 (0.545) −0.677 (0.707)	0.124 −0.074
No. of chronic diseases (Ref. = 1) 2 3	1.222 (0.545) 1.698 (0.701)	0.175 * 0.187 *
Physical disability	0.087 (0.028)	0.225 **
Interpersonal trust	−0.924 (0.396)	−0.165 *
Reciprocity	−1.087 (0.296)	−0.264 **
R^2^ F	0.380 5.738 **	

SE = standard error, Ref = reference, * = *p* ˂ 0.05, ** = *p* ˂ 0.01.

**Table 4:** Mediation effect based on PROCESS model 4.

Variables	B (SE)	LLCI	ULCI
Outcome: Depressive symptoms			
Physical disability	0.097 ** (0.029)	0.037	0.156
Average monthly income (PKR) (Ref. ≤15,000)			
15,001–30,000	0.972 (0.584)	−0.182	2.127
>30,000	−1.224 (0.758)	−2.723	0.274
Living status	−1.484 ** (0.533)	−2.538	−0.431
R^2^ F	0.155 6.510*		
Outcome: Depressive symptoms			
Interpersonal trust	−0.884 * (0.425)	−1.726	−0.042
Reciprocity	−0.883 ** (0.315)	−1.507	−0.259
Physical disability	0.078 ** (0.029)	0.019	0.136
Average monthly income (PKR) (Ref. ≤15,000)			
15,001–30,000	1.020 (0.565)	−0.097	2.137
>30,000	−1.051 (0.739)	−2.513	0.411
Living status	−1.438 ** (0.517)	−2.461	−0.415
R^2^ F	0.224 6.715 *		

SE = standard error, LLCI = lower level confidence interval, ULCI = upper level confidence interval, Ref = reference, * = *p* ˂ 0.05, ** = *p* ˂ 0.01.
